# Neonatal White Matter Microstructure and Emotional Development during the Preschool Years in Children Who Were Born Very Preterm

**DOI:** 10.1523/ENEURO.0546-20.2021

**Published:** 2021-09-29

**Authors:** Dana Kanel, Lucy D. Vanes, Diliana Pecheva, Laila Hadaya, Shona Falconer, Serena J. Counsell, David A. Edwards, Chiara Nosarti

**Affiliations:** 1 Centre for the Developing Brain, School of Biomedical Engineering and Imaging Sciences, King’s College London, London SE1 7EH, United Kingdom; 2 Department of Child and Adolescent Psychiatry, Institute of Psychiatry, Psychology and Neuroscience, King’s College London, London SE5 8AF, United Kingdom; 3 MRC Centre for Neurodevelopmental Disorders, King’s College London, London SE1 1UL, United Kingdom

**Keywords:** diffusion MRI, preterm children, socio-emotional development, tractography

## Abstract

Children born very preterm (<33 weeks of gestation) are at a higher risk of developing socio-emotional difficulties compared with those born at term. In this longitudinal study, we tested the hypothesis that diffusion characteristics of white matter (WM) tracts implicated in socio-emotional processing assessed in the neonatal period are associated with socio-emotional development in 151 very preterm children previously enrolled into the Evaluation of Preterm Imaging study (EudraCT 2009-011602-42). All children underwent diffusion tensor imaging at term-equivalent age and fractional anisotropy (FA) was quantified in the uncinate fasciculus (UF), inferior fronto-occipital fasciculus (IFOF), inferior longitudinal fasciculus (ILF), and superior longitudinal fasciculus (SLF). Children’s socio-emotional development was evaluated at preschool age (median = 4.63 years). Exploratory factor analysis conducted on the outcome variables revealed a three-factor structure, with latent constructs summarized as: “emotion moderation,” “social function,” and “empathy.” Results of linear regression analyses, adjusting for full-scale IQ and clinical and socio-demographic variables, showed an association between lower FA in the right UF and higher “emotion moderation” scores (β = −0.280; *p *<* *0.001), which was mainly driven by negative affectivity scores (β = −0.281; *p *=* *0.001). Results further showed an association between higher full-scale IQ and better social functioning (β = −0.334, *p *<* *0.001). Girls had higher empathy scores than boys (β = −0.341, *p *=* *0.006). These findings suggest that early alterations of diffusion characteristics of the UF could represent a biological substrate underlying the link between very preterm birth and emotional dysregulation in childhood and beyond.

## Significance Statement

Children born very preterm are at a higher risk of developing socio-emotional difficulties compared with those born at term. Our study showed that early alterations of diffusion characteristics of the uncinate fasciculus (UF) in very preterm infants assessed at term-equivalent age were associated with emotional dysregulation in childhood. The identification of early biological substrates linked to emotional development could create opportunities for the prevention and targeting of emerging emotional problems to enhance children’s mental health.

## Introduction

Children who were born very preterm (<33 weeks of gestation) are at heightened risk of experiencing socio-emotional difficulties, which include diminished social competence and reduced ability to self-regulate their emotions and behaviors (Spittle et al., 2009; Jones et al., 2013; Witt et al., 2014; Montagna and Nosarti, 2016). Socio-emotional difficulties in childhood have been associated with the later emergence of psychiatric symptoms (Woodward et al., 2017; Thomson et al., 2019).

Our current understanding of the etiology of socio-emotional difficulties associated with very preterm birth is limited. Possible underlying causes include altered neurodevelopment, implicating brain structural and functional connectivity (Counsell et al., 2014; Duerden et al., 2015; Keunen et al., 2017; Dimitrova et al., 2020). However, studies directly investigating the association between brain alterations and children’s socio-emotional outcomes following very preterm birth are scarce (Fischi-Gómez et al., 2015; Mossad et al., 2017; Urbain et al., 2019). Only a few investigations to date have used a longitudinal design to identify neural features present in the neonatal period that are associated with later socio-emotional problems (Rogers et al., 2012), and studies have predominantly focused on resting state functional connectivity. These studies have suggested associations between alterations in neonatal functional amygdala connectivity and internalizing symptoms, and between alterations in ventral attention-default mode network connectivity and behavioral inhibition in preterm born toddlers (Rogers et al., 2017; Sylvester et al., 2018). Given that functional connectivity is constrained by the anatomic structure of the human cerebral cortex (Honey et al., 2009), investigating relationships between neonatal white matter (WM) diffusion characteristics and later socio-emotional outcomes may further add to our understanding of how the brain’s emerging architecture contributes to shaping very preterm children’s development.

Socio-emotional processing is underpinned by integrated activity across an extended socio-emotional network (Catani et al., 2013). Therefore, structural connectivity alterations within this system may represent a neural substrate of social-emotional impairments. Emotional disorders have been characterized by WM diffusion characteristic alterations in several tracts, including the uncinate fasciculus (UF), which connects the temporo-amygdala-orbitofrontal network, the inferior fronto-occipital fasciculus (IFOF), which connects the dorsolateral and inferolateral frontal cortex with the occipital and posterior temporal cortex, the inferior longitudinal fasciculus (ILF), which links occipital and temporal lobes, and the superior longitudinal fasciculus (SLF), which connects parietal to frontal cortical regions (Jenkins et al., 2016; Wang et al., 2016).

These different tracts have been studied in relation to various behavioral outcomes, such as memory and cognition (Riley et al., 2010; Chen et al., 2020; Koshiyama et al., 2020), but also to specific aspects of socio-emotional processing: the UF with emotion regulation (Von Der Heide et al., 2013; Eden et al., 2015), the IFOF and ILF with the ability to decode human facial emotions (Philippi et al., 2009; Unger et al., 2016), and the SLF with emotional empathy (Parkinson and Wheatley, 2014).

Differences in the diffusion characteristics of these tracts between very preterm and term-born individuals have also been investigated, with previous studies showing inconsistent results, such as reduced fractional anisotropy (FA) values in the UF (Mullen et al., 2011; Travis et al., 2015; Vollmer et al., 2017; Young et al., 2018), both increased (Dodson et al., 2017) and decreased FA values in the IFOF (Salvan et al., 2014; Vollmer et al., 2017; Young et al., 2018), decreased FA values in the ILF (Travis et al., 2015; Vollmer et al., 2017; Young et al., 2018) and SLF (Young et al., 2018), and no group differences in the SLF (Vollmer et al., 2017). Further, very preterm infants and children also display increased diffusivities when compared with term-born controls, including mean diffusivity (MD), radial diffusivity (RD), and axial diffusivity (AD; Young et al., 2018; Lautarescu et al., 2020; Brenner et al., 2021).

The current longitudinal investigation aimed to test the hypothesis that diffusion characteristics of WM tracts implicated in socio-emotional processing assessed at term equivalent age are associated with socio-emotional development during the preschool years in children who were born very preterm.

## Materials and Methods

### Participants

A total of 511 participants were enrolled into the Evaluation of Preterm Imaging study (ePrime, (EudraCT 2009-011602-42); Edwards et al., 2018). They were recruited at birth in 2010–2013 from hospitals within the North and Southwest London Perinatal Network. Inclusion criteria were birth before 33 weeks of gestational age (GA) and maternal age over 16 years. Exclusion criteria included the presence of major congenital malformation, prior magnetic resonance imaging (MRI), metallic implants, parents unable to speak English, or being subject to child protection proceedings. Infants underwent MRI at term-equivalent age, defined as 38–44 weeks of GA (mean = 42.24; SD = 1.41). At four to seven years of age, children were invited to the Centre for the Developing Brain, St Thomas’ Hospital, London, for a neurodevelopmental assessment. Invitations for follow-up were sent in chronological order of birth to all children who were past their fourth birthday. The study closed on September 1, 2019. Written informed consent was obtained from participants’ carer(s) following procedures approved by the Stanmore Research Ethics Committee (14/LO/0677). The study was conducted in accordance with the Code of Ethics of the World Medical Association (Declaration of Helsinki).

**Figure 1.**
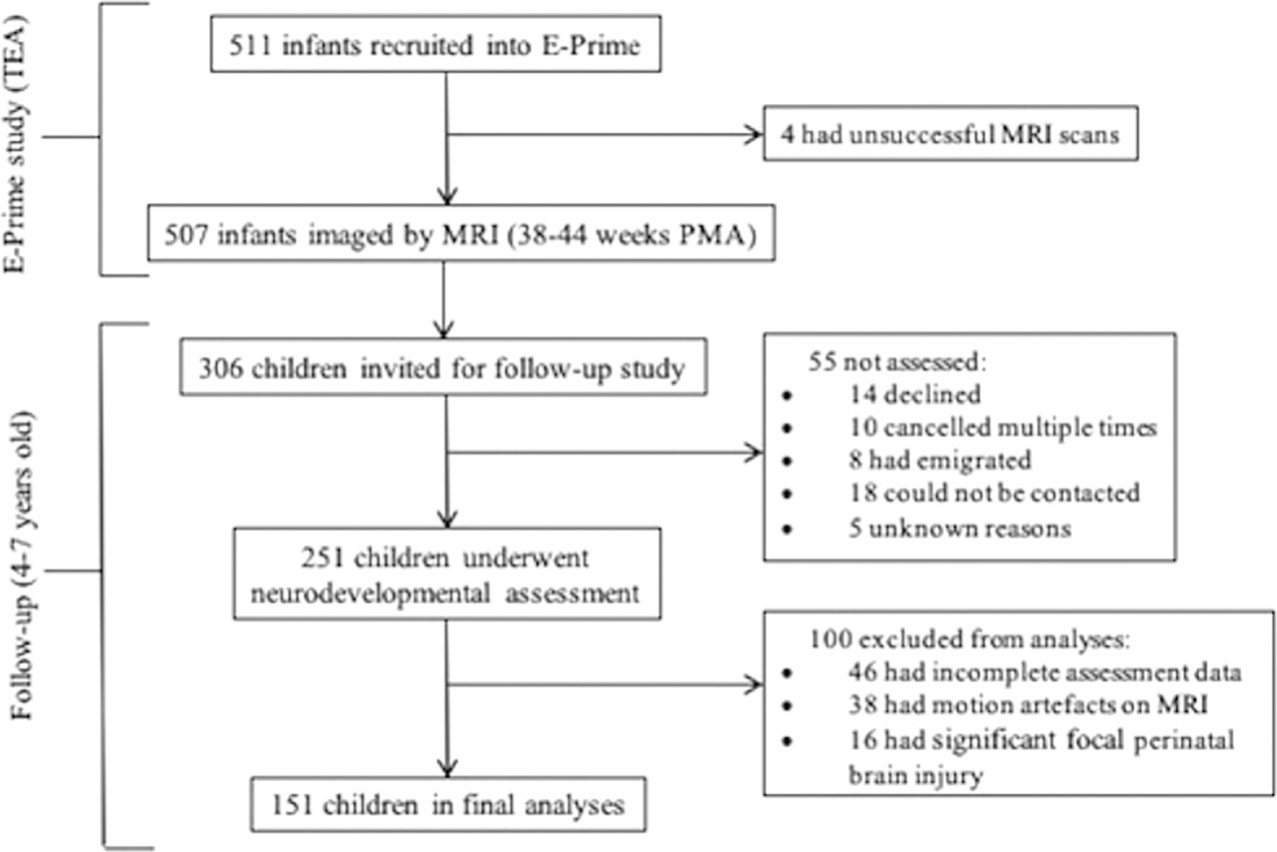
Recruitment flowchart. TEA, term-equivalent age; PMA, postmenstrual age.

Figure 1 provides detailed recruitment information. To summarize, 55 children invited for follow-up study were not assessed at four to seven years for the reasons listed in Figure 1. A further one hundred children were excluded from the final analyses, because of incomplete assessment data (*n* = 46), motion artefacts on MRI (*n* = 38) or significant focal perinatal brain injury (*n* = 16), defined as periventricular leukomalacia, hemorrhagic parenchymal infarction and other ischemic or hemorrhagic lesions (Barnett et al., 2018), but not including punctate lesions or diffuse excessive high signal in WM on T2-weighted images. The final sample consisted of 151 very preterm born participants who had T1-weighted and T2-weighted MRI and diffusion MRI (dMRI) at 38–44 weeks of term-equivalent and subsequently participated in the follow-up assessment at four to seven years.

### Procedure

#### Perinatal clinical and socio-demographic data

Perinatal clinical and socio-demographic data were collected, with permission, from the Standardized Electronic Neonatal Database. Index of Multiple Deprivation (IMD) score was computed from the postcode of the parent at the time of infant birth (Department for Communities and Local Government, 2011; https://tools.npeu.ox.ac.uk/imd/) and provided a proxy for family socio-economic status. The IMD measures social risk by comparing each neighborhood to all others in the country and is based on seven domains of deprivation (with varying weighting, as follows): income (22.5%), employment (22.5%), education skills and training (13.5%), health and disability (13.5%), barriers to housing and services (9.3%), living environment (9.3%), and crime (9.3%). Maternal education was defined as age on leaving full-time education, split into two groups (1) at or before 19 years; (2) after 19 years (Kleine et al., 2020).

#### MRI acquisition and analysis

A pediatrician experienced in MRI procedures supervised all MR imaging. Pulse oximetry, temperature, and electrocardiography data were monitored throughout the session. Silicone-based putty (President Putty, Coltene Whaledent), as well as neonatal earmuffs (MiniMuffs, Natus Medical Inc.), were used for ear protection. Oral chloral hydrate (25–50 mg kg^−1^) was administered to infants whose parents chose sedation for the procedure.

MR imaging was performed on a 3-Tesla system (Philips Medical Systems) sited on the neonatal intensive care unit using an eight-channel phased array head coil. High-resolution anatomic images were acquired with pulse sequence parameters: T2-weighted fast-spin echo imaging: TR = 8670 ms, TE = 160 ms, flip angle 90°, slice thickness 2 mm with 1-mm overlapping slices, in-plane resolution 0.86 × 0.86 mm. dMRI data were acquired in the transverse plane in 32 non-collinear directions with the following parameters: TR = 8000 ms, TE = 49 ms, voxel size: 2 mm isotropic, b value: 750 s/mm^2^, sense factor of 2, 1 non-diffusion-weighted image, *b *=* *0.

Diffusion-weighted images were visually inspected in three orthogonal planes for the presence of motion artifact and corrupt diffusion-weighted volumes were excluded before tensor fitting. All participants included in analyses had five or fewer excluded volumes. Non-brain tissue was removed using BET (version 2.1; http://fsl.fmrib.ox.ac.uk/fsl/fslwiki/BET; Smith, 2002; Jenkinson et al., 2012), images were corrected for eddy current artefacts using *eddy_correct* (Andersson and Sotiropoulos, 2016), and tensor model was fitted using *dtifit* from FSL (FMRIB; http://fsl.fmrib.ox.ac.uk).

Tract-specific analysis (TSA; Yushkevich et al., 2008) was used to derive dMRI measures for selected WM tracts, as described in detail in Pecheva et al. (2017). TSA is a WM analysis method that creates skeleton models of individual WM tracts onto which diffusion data can be projected for statistical analysis. All subjects were registered to a study-specific template using a tensor-based algorithm (Zhang et al., 2006). Following registration, tracts of interest were delineated from the template using deterministic tractography based on the FACT approach (Mori et al., 1999). Whole-brain tractography was seeded from a WM mask, defined by thresholding the template FA map at 0.1, and regions of interest were drawn manually according to the protocol described previously (Wakana et al., 2007), by a single rater (D.P.; Table 1). The following tracking parameters were used: maximum angle threshold of 45°, step size of 0.5 mm and minimum FA threshold of 0.1. From the tractography, a surface skeleton representation with clearly-defined tract boundaries was derived for each WM pathway. TSA samples data to be projected onto each point of the skeleton by searching for the maximum FA value along the unit normal from that point to the tract boundary. This is done for each subject. The data projection step serves two main purposes. First, it is a dimensionality reduction step which increases sensitivity, similar to smoothing (Yushkevich et al., 2008). Furthermore, as tract FA values tend to be higher in the center of a tract, projecting the maximum FA value accounts for residual misalignments and improves inter-subject correspondence by forcing a comparison between tract centers across subjects (Smith et al., 2006).

**Table 1:** Definitions of ROIs drawn manually to delineate WM tracts

Tract	Inclusion ROI 1	Inclusion ROI 2	Exclusion ROI
UF	Entire temporal lobe identified in coronal plane at level where frontal and temporal lobe are no longer connected	All projections into frontal lobe	Fibers which project into anterior limb of external capsule and posteriorly
IFOF	Occipital lobe selected in coronal plane identified halfway between posterior edge of cingulum and posterior of the brain	Entire hemisphere in coronal plane at level of the genu of CC identified in mid-sagittal slice	Fibers crossing medially through anterior commissure
ILF	Entire hemisphere selected in coronal plane at posterior edge of cingulum identified at mid-sagittal slice	Entire temporal lobe identified in the coronal plane at level where frontal and temporal lobe are no longer connected	Fibers that track medially into fornix and CC
SLF	Identified in coronal plane at lowest axial level in which fornix can be identified as a single structure	Projections passing through coronal plane at level of splenium of CC identified in mid-sagittal slice	Fiber that project into external capsule

UF, uncinate fasciculus; IFOF, inferior fronto-occipital fasciculus; ILF, inferior longitudinal fasciculus; SLF, superior longitudinal fasciculus; CC, corpus callosum.

**Figure 2.**
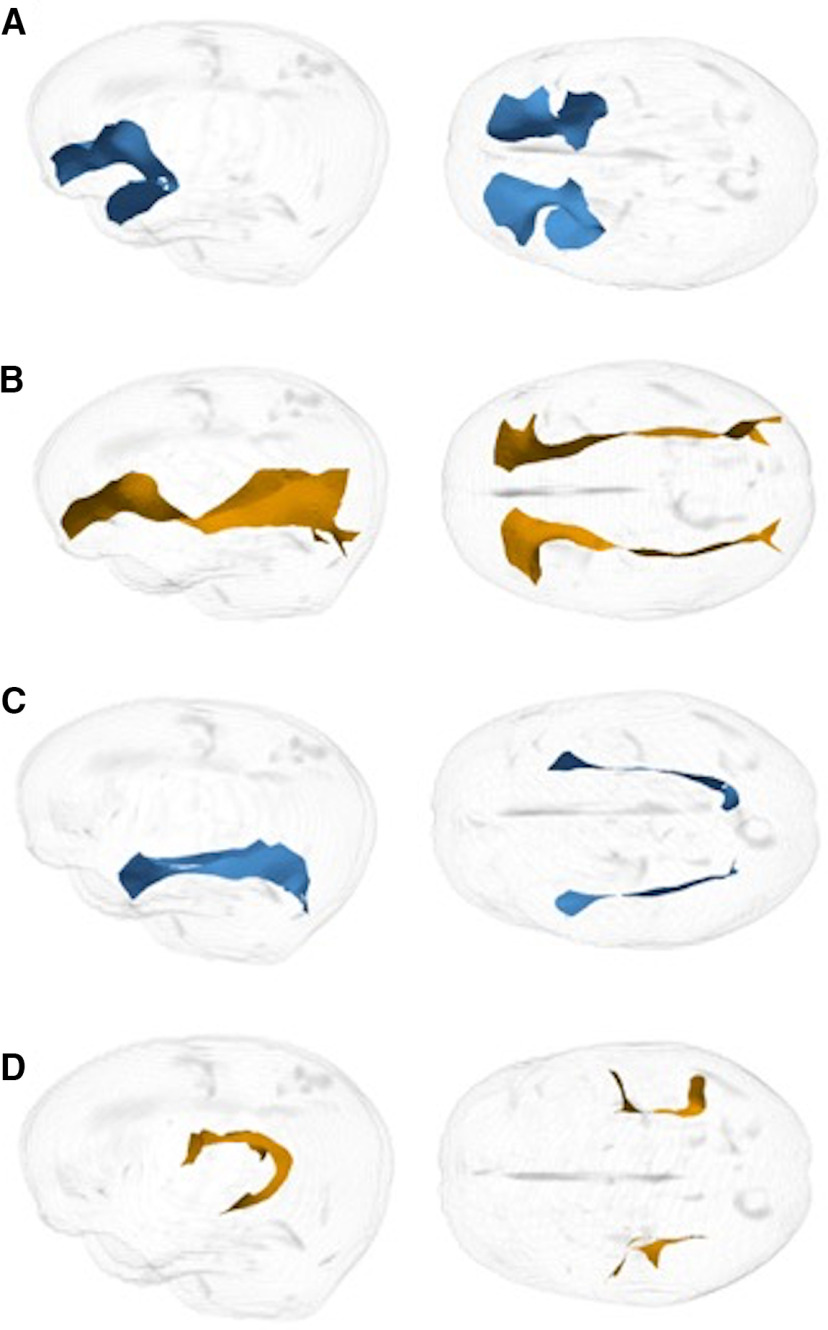
Example of right sagittal and bilateral axial surface representation of tracts. ***A***, UF. ***B***, IFOF. ***C***, ILF. ***D***, SLF.

From the tractography results, a medial surface was determined for the UF, IFOF, ILF, and SLF. The medial surface simultaneously defined the tract skeleton and boundary (Yushkevich and Zhang, 2013). Diffusion data from every subject was then projected onto the skeleton. TSA sampled data to be projected onto each point of the skeleton by searching along the unit normal from that point to the tract boundary (Pecheva et al., 2017). FA values were calculated for each tract. Examples of surface representation of tracts are shown in Figure 2.

#### Neurodevelopmental outcomes

The Wechsler Preschool and Primary Scale of Intelligence (WPPSI-IV; Wechsler, 2012) was used to estimate children’s full-scale IQ. Parents completed the Strengths and Difficulties Questionnaire (SDQ; Goodman, 1997), a behavioral screening measure for general childhood psychopathology, comprising 25 items categorized into five subscales: emotional symptoms, conduct problems, hyperactivity/inattention, peer relationship problems and prosocial behavior.; the Children’s Behaviour Questionnaire–Very Short Form (CBQ; Putnam and Rothbart, 2006) which assesses the child’s temperament using 36 items summarized into three broad scales (negative affectivity, effortful control and surgency); the Empathy Questionnaire (EmQue; Rieffe et al., 2010), a 20 item questionnaire which measures empathy-related behaviors in young children, summarized into three scales: emotion contagion, attention to others’ emotions and prosocial responses to others’ emotions; and the Social Responsiveness Scale (SRS-2; Constantino and Gruber, 2012), an assessment of social impairments associated with autism-spectrum behaviors, which provides subscale scores for social awareness, social cognition, social communication, social motivation, restricted interests and repetitive behaviors.

To measure facial emotion recognition abilities, a new task was created based on (Gao and Maurer, 2009), which used static stimuli from the validated Dartmouth database of children’s faces (Dalrymple et al., 2013). Four boys and four girls were chosen from the database and 6 emotions (happy, surprise, fear, anger, disgust and sadness) plus neutral faces were used. The task consisted of two testing blocks, whereby the first of these included pictures showing happy, surprised and fearful expressions, and the second contained pictures showing sad, disgusted and angry expressions, with both blocks including neutral faces. Stimuli were presented singly, and participants were asked to identify which emotion each image was representing. Accuracy was measured based on the correct identification of emotions. Male and female models were allocated evenly between the emotion and neutral expressions. For every emotion, two levels of intensity were created by morphing a neutral face with the emotional face of the same model, to create, for example, 50% and 100% happy faces. The use of different intensities has been shown to promote the detection of subtle differences in abilities to recognize emotions (Horning et al., 2012; Kessels et al., 2014). Fantamorph software was used to create these morphed images (http://www.fantamorph.com/index.html), by manually positioning points on the anatomic landmarks in the photograph of each face. Distortions caused by the morphing process in the eye and mouth regions were edited using Photoshop. In total, there were 56 stimuli [(2 intensity levels × 6 emotions × 4 models) + (2 neutral expressions × 4 models)]. The task created here has no working memory load, and therefore only participant’s accuracy is measured. The total number of correct responses the child made was added to produce an emotion recognition score.

Lower accuracy scores on the emotion recognition task correlated with higher SRS-2 T-scores, indicating increased social difficulties [social information processing (*r* = −0.261; *p *<* *0.001), social communication (*r* = −0.201; *p *=* *0.003), social motivation (*r* = −0.201; *p *=* *0.003), restricted interests and repetitive behavior (*r* = −0.228; *p *=* *0.001), social communication index (SCI; *r* = −0.234; *p *=* *0.001), and total score (*r* = −0.237; *p *<* *0.001)]. Crucially, the task was most strongly associated with social information processing, suggesting it successfully measured the key construct it was designed to, in pediatric samples.

### Statistical analyses

Twenty-eight perinatal clinical variables obtained from all ePrime participants (*n* = 511) were summarized using principal component analysis (PCA) applying Promax rotation, using SPSS 26. Maternal variables were: preeclampsia and pregnancy induced hypertension, antenatal hypertension, placental abruption or antenatal hemorrhage, premature rupture of membranes, urinary tract infection, gestational diabetes, oligohydramnios, polyhydramnios, drug abuse, in vitro fertilization, bacterial infection (all y/n), and mode of delivery (vaginal/elective/emergency). Infant variables were: sex, GA (weeks and days), birth weight (grams), multiple pregnancy (singleton/multiple), antenatal steroid administration (no/partial/full course), twin-to-twin transfusion, chorioamnionitis, intrauterine growth restriction, surfactant administration, treatment for patent ductus arteriosus, surgical treatment for necrotizing enterocolitis, formula feeding, feeding on maternal expressed breast milk (all y/n), days on mechanical ventilation, days on continuous positive airway pressure (cPAP), and days on parenteral nutrition (TPN). All variables were coded so that higher values reflected greater clinical risk. Communalities were checked, and as all were above 0.2, no items were removed (Costello and Osborne, 2005).

R using R studio was used to perform all following analyses, using a non-random experimental design. Maximum-likelihood factor analysis with Varimax rotation was performed using the stats v3.6.2 package. Factor analysis included the following outcome variables: four SDQ subscales (emotional symptoms, conduct problems, peer relationship problems and prosocial behavior), three CBQ subscales (negative affectivity, surgency and effortful control), three EmQue subscales (emotion contagion, attention to others’ feelings and prosocial actions); the SRS-2 SCI and accuracy on the emotion recognition task. All neurodevelopmental subscale scores were standardized. Factors were extracted based on the criterion of having eigenvalues >1 and examination of scree plots. Factor scores were then used in subsequent analyses.

In order to find the best predictors of children’s socio-emotional outcomes, best-fit linear models were selected using an automated model selection process, using the *glmulti* package (Calcagno and de Mazancourt, 2010). Before model selection, multicollinearity between the chosen tracts was assessed by calculating a variance inflation factor (VIF) for each tract, which consists of comparing the overall model variance to the variance of a model that includes only that single independent tract. Two variables with VIF ≥ 10 were excluded from subsequent analyses (Hair, 2009), i.e., FA values of the left and right IFOF. For each socio-emotional outcome (emotion moderation, social function, and empathy), model comparison was performed using Akaike Information Criterion (AIC; Akaike, 1974) and 8450 models were compared. Variables included in each model were: FA values of the left UF, ILF and SLF; right UF, ILF and SLF; postmenstrual age at scan (PMA), corrected age at follow-up assessment, IQ, IMD, maternal education, sex, and neonatal sickness index. Linear regression was then performed to study the association between predictor variables included in the best-fit model and socio-emotional factor scores.

## Results

Participants’ characteristics are summarized in Table 2. Results of PCA performed to summarize perinatal clinical variables showed one factor that explained 72% of their variance. The Kaiser–Meyer–Olkin measure of sampling adequacy was 0.73, Bartlett’s test of sphericity was significant (χ^2^ = 3597.79, *p < *0.01), and all factor loadings were above 0.2. Factor weights were between 0.767 and 0.898 for GA, days on TPN, days on cPAP, days on mechanical ventilation and surfactant administration. This factor was labeled as “neonatal sickness index.” Internal reliability assessed using Cronbach’s α was good (α = 0.86).

**Table 2:** Participants’ clinical and socio-demographic characteristics

GA at birth (weeks), median (IQR)	30.29 (28.79–31.79)		
Birth weight (g), median (IQR)	1275 (980–1570)		
PMA at MRI (weeks), mean (±SD)	42.22 (0.79)		
CA at assessment, median (IQR)	4.63 (4.1−5.16)		
Female, number (percentage)	71 (47%)		
IQ score, mean (±SD)	108.03 (17.00)		
IMD score quintiles	1 (least deprived)	36	23.8%
	2	26	17.2%
	3	37	24.5%
	4	35	23.2%
	5 (most deprived)	17	11.3%
Maternal education ≥19 years, number (percentage)	117 (77.5%)		

CA, corrected age at assessment; GA, gestational age; IMD, index of multiple deprivation; IQR, interquartile range; MRI, magnetic resonance imaging; PMA, postmenstrual age at neonatal MRI.

Children who were included in the final analyses had lower neonatal sickness index scores (*t* = 2.721, *p *=* *0.007) and higher IQ scores (*t* = −3.449, *p *=* *0.001) compared with those who were not included. Results were similar after exclusion of non-participating children with significant focal perinatal brain injury on neonatal MRI (neonatal sickness index score: *t* = 2.903, *p *=* *0.004; IQ: *t* = −2.752, *p *=* *0.006).

### Socio-emotional outcomes

Descriptive statistics for children’s socio-emotional outcome measures are shown in Table 3.

**Table 3:** Descriptive statistics for children’s outcome measures

SDQ emotional symptoms, median (IQR)	1 (0–3)
SDQ conduct problems, median (IQR)	1 (0–3)
SDQ peer relationship problems, median (IQR)	1 (0–2)
SDQ prosocial behavior, median (IQR)	8 (6–9)
CBQ negative affectivity, mean (±SD)	4.284 (0.659)
CBQ surgency, mean (±SD)	5.133 (0.641)
CBQ effortful control, mean (±SD)	4.494 (0.485)
EmQue emotion contagion, median (IQR)	0.17 (0–0.5)
EmQue attention to others’ feelings, mean (±SD)	1.299 (0.381)
EmQue prosocial, mean (±SD)	1.076 (0.399)
SRS SCI, mean (±SD)	47.037 (8.169)

Raw values are shown. CBQ, children’s behavior questionnaire; EmQue, empathy questionnaire; IQR, interquartile range; SDQ, strengths and difficulties questionnaire; SRS, social responsiveness scale.

**Figure 3.**
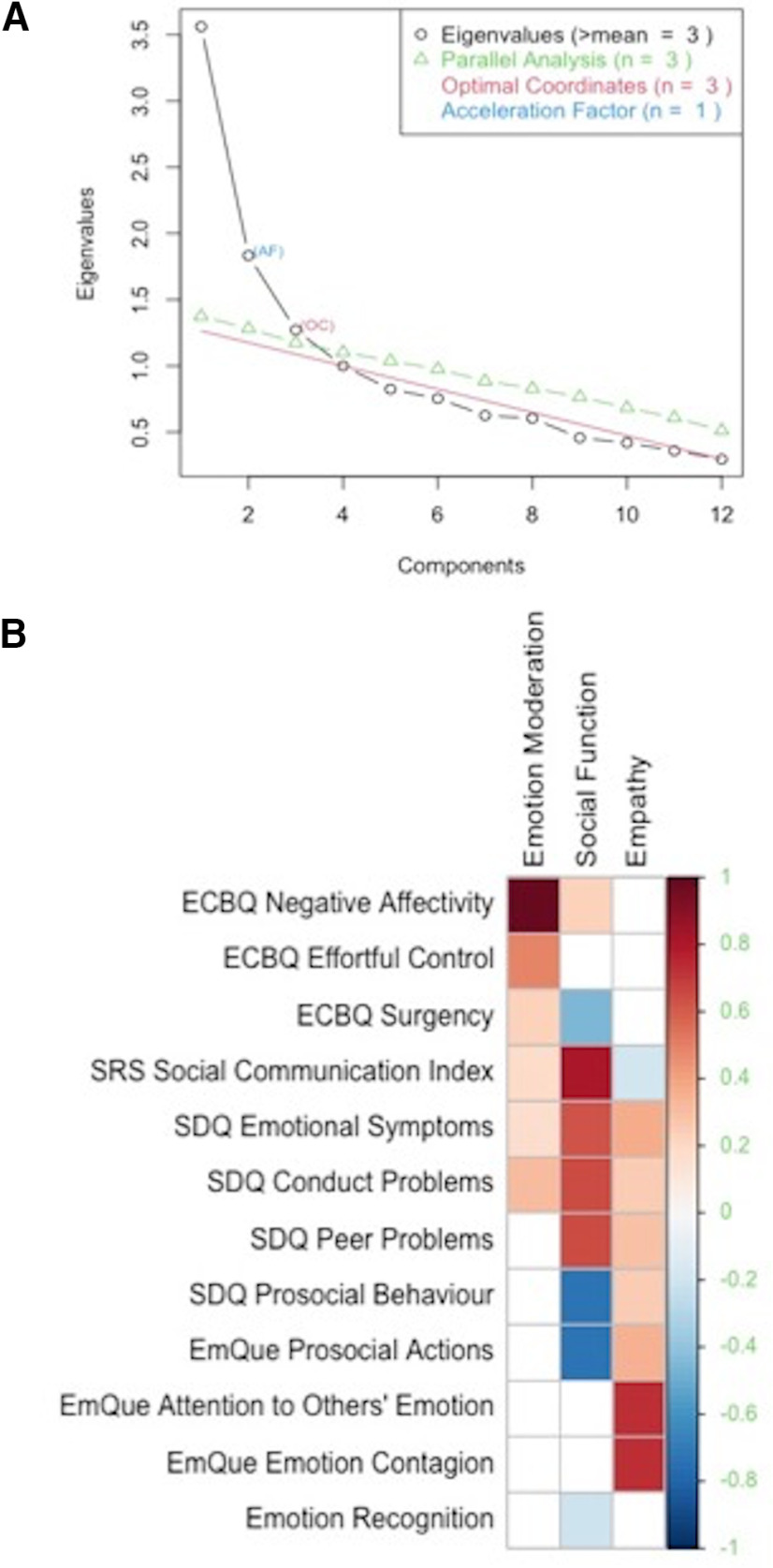
***A***, Scree plot representing eigenvalues, used to determine number of factors to retain. ***B***, Heatmap indicating factor loadings on emotion moderation, social function, and empathy.

Exploratory factor analysis conducted on the 12 socio-emotional outcomes showed a three-factor structure. The Kaiser–Meyer–Olkin measure of sampling adequacy was 0.76, Bartlett’s test of sphericity was significant (χ^2^ = 469.977, *p *<* *0.001), and all factor loadings were above 0.4. The three latent constructs accounted for 42% of the variance in socio-emotional outcomes and are graphically displayed in Figure 3. These are summarized as: factor 1 or “emotion moderation,” characterized by positive loadings (between 0.48 and 0.93) for CBQ negative affectivity and CBQ effortful control scores. Factor 2 or “social function,” which loads onto higher SDQ emotional symptoms, SDQ conduct problems, SDQ peer relationship problems scores and SRS-2 SCI; as well as lower scores on SDQ prosocial behavior, EmQue prosocial actions and CBQ surgency (between –0.67 and 0.74), indicating more emotional and behavior problems, heightened antisocial behavior and more socializing difficulties. Factor 3 or “empathy” is defined by positive loadings for EmQue emotion contagion and EmQue attention to others’ scores (both 0.56), indicating a higher degree of empathic displays. Emotion recognition scores did not substantially load onto any of the factors.

### Diffusion properties of WM tracts

Mean FA values of the UF, IFOF, ILF and SLF, are shown in Table 4.

**Table 4:** Descriptive statistics for diffusion properties of WM tracts implicated in emotion processing

Tract	Left FA, mean (±SD)	Right FA, mean (±SD)
UF	0.165 (0.015)	0.169 (0.015)
IFOF	0.209 (0.019)	0.207 (0.017)
ILF	0.207 (0.021)	0.198 (0.019)
SLF	0.162 (0.014)	0.188 (0.016)

UF, uncinate fasciculus; IFOF, inferior fronto-occipital fasciculus; ILF, inferior longitudinal fasciculus; SLF, superior longitudinal fasciculus.

### Best-fit predictors of socio-emotional outcomes

**Figure 4.**
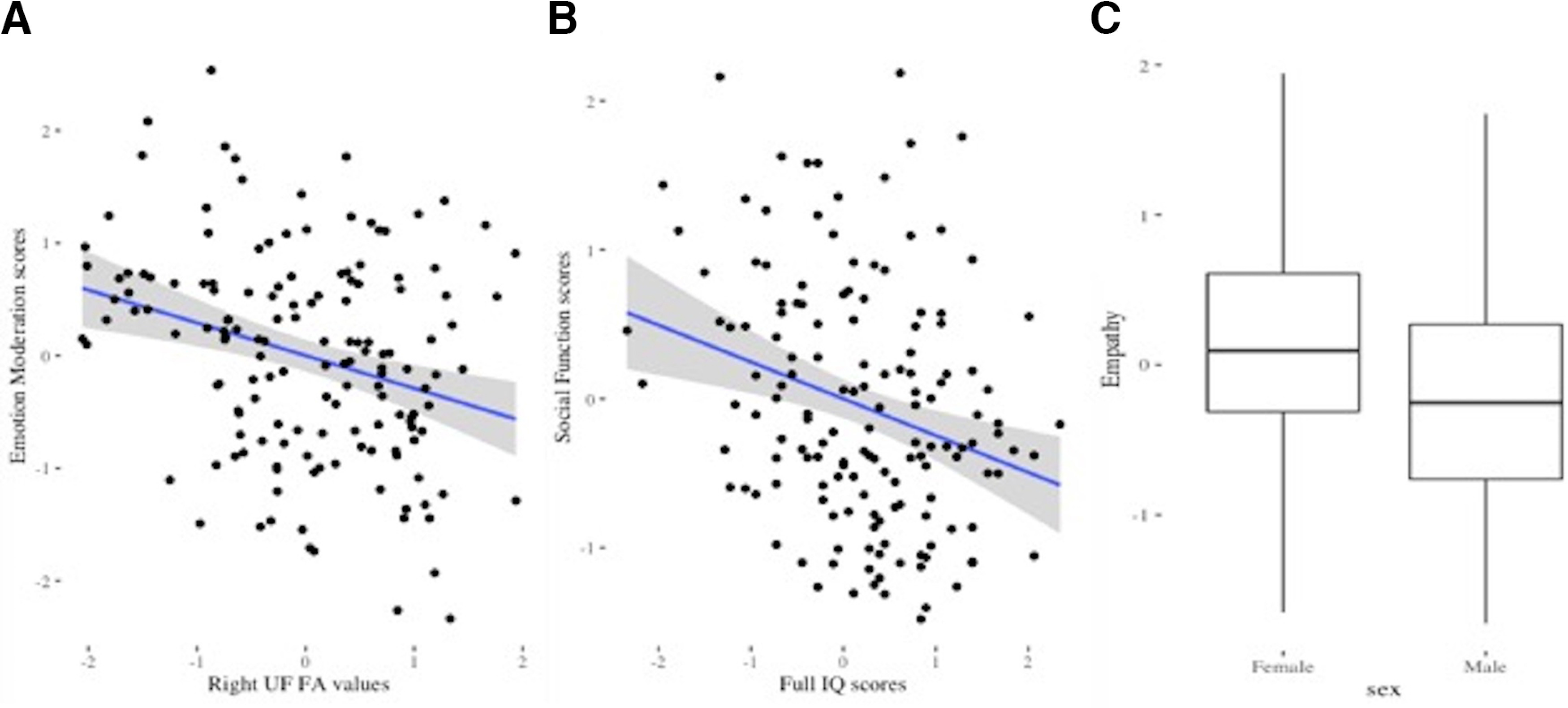
***A***, Scatterplot showing negative relationship between right UF FA values and emotion moderation scores (one outlier removed, total *n* = 150). ***B***, Scatterplot showing negative relationship between IQ scores and social function scores (one outlier removed, total *n* = 150). ***C***, Boxplot showing sex differences in empathy scores. Outliers were defined as values >1.5 times the value of the interquartile range beyond the quartiles. All values were scaled prior to analyses.

Best-fit predictors of emotion moderation were right UF FA values and full-scale IQ (AIC value = 387.387). Lower FA values in the right UF were associated with higher emotion moderation scores (β = −0.280, *p *<* *0.001; Fig. 4). In order to aid interpretation, *post hoc* analyses decomposed the emotion moderation factor into the two variables that loaded onto it (CBQ negative affectivity and CBQ effortful control scores). Results showed that the association between right UF FA values and emotion moderation was mainly driven by their relationship to negative affectivity scores (β = −0.281; *p *=* *0.001). UF FA values were not significantly associated with effortful control scores (β = −0.126; *p *=* *0.150).

Best-fit predictors of social function were sex, corrected age at assessment and full-scale IQ (AIC value = 407.210). Higher full-scale IQ was associated with better social functioning (i.e., lower social function factor scores; β = −0.334, *p *<* *0.001). Best-fit predictor of empathy was sex and IMD (AIC value = 346.983). Girls had higher empathy scores than boys (β = −0.319, *p *=* *0.006). FA values of the WM tracts implicated in emotion processing were not associated with social function and empathy.

Results of regression analyses for the three outcome models (emotion moderation, social function, and empathy) including best-fit predictors remained significant after applying Bonferroni correction, correcting for the six WM tracts (adjusted *p* value for significance = 0.008; Table 5).

**Table 5:** Results from regression analyses for socio-emotional factors including best-fit predictors

Outcome	Data structure	Predicting variable	β	*p* value
Emotion moderation	Normal distribution	Right UF FA	−0.280	<0.001^*^
		Full-scale IQ	−0.129	0.104
Social function	Normal distribution	Sex	0.279	0.048
		Corrected age at assessment	−0.103	0.138
		Full-scale IQ	−0.334	<0.001^*^
Empathy	Normal distribution	Sex	−0.341	0.006^*^
		IMD	−0.110	0.083

* Analyses significant after applying Bonferroni correction (adjusted *p* value for significance = 0.008).

## Discussion

This study found that WM diffusion characteristics in the UF assessed at term equivalent age in very preterm infants were associated with childhood emotion moderation scores, which summarize a latent factor reflecting both increased negative affectivity and enhanced effortful control. Negative affectivity refers to a reactive temperamental trait that is characterized by an overall negative outlook of oneself and the surrounding world (e.g., frustration, anger, sadness). Effortful control is conceptualized as an umbrella term referring to a child’s capacity to focus and shift attention, intentionally inhibit a response, and respond to low-intensity stimulation and reward (Rothbart et al., 2003). The two traits, negative affectivity and effortful control, could be compared with constructs used to describe adult personality, neuroticism and constraint/conscientiousness, respectively (Digman, 1990). Summary scores for negative affectivity and effortful control tend to be negatively correlated in community samples (Putnam et al., 2006), hence the factor described here as emotion moderation, which combines high negative affectivity and enhanced effortful control, may look unintuitive at first. This factor could be interpreted as reflecting an adaptive strategy, whereby children use regulatory skills to moderate the impact of reactive systems, i.e., negative emotionality (Rothbart and Bates, 1998; Nigg, 2006). In line with this hypothesis, Eisenberg and colleagues showed that negative emotionality and low regulation were maladaptive, (Eisenberg et al., 2001) while Belsky et al. (2001) found that high levels of orienting/effortful control moderated the impact of children’s negative affectivity on behavioral outcomes.

Lower FA values in the right UF assessed at term were associated with higher emotion moderation scores in childhood. When this factor was decomposed into the two variables that loaded onto it (negative affectivity and effortful control) we found that this association was driven by the relationship between right UF FA values and negative affectivity. Anatomically, the UF is sited withing the limbic system, and connects the “temporo-amygdala-orbitofrontal network” (Catani et al., 2013), which has been suggested to play a pivotal role in “affective tagging” (Von Der Heide et al., 2013), i.e., the assignment of emotional tone, such as positive and negative feelings, to the representations stored in the anterior temporal lobe (Olson et al., 2013). Diffusion characteristics of the UF have been previously implicated in a variety of psychiatric disorders (Von Der Heide et al., 2013) including anxiety disorder (Phan et al., 2009) and major depression (Cullen et al., 2010) and have also been associated with early adversity and future psychological vulnerability to stress (Hanson et al., 2015). A study in major depression investigating diffusion characteristics and resting state functional connectivity of the UF showed structural alterations together with functional orbitofrontal cortex-amygdala inhibition, suggesting this dysconnectivity pattern was mediated by “top‐down” influences from the frontal cortex to the amygdala (Zheng et al., 2018). We speculate that early diffusion characteristics of the UF may be associated with difficulties in top-down regulation, leading to children’s inability to down-regulate amygdalar activity, resulting in the tendency to attribute negative feelings to certain contexts and situations, and in the overexpression of negative affect.

Furthermore, although our results did not show a direct association between UF FA values and effortful control, and the UF has not been selectively implicated in effortful/cognitive control in the published literature (Noble et al., 2013), it may nevertheless be relevant to effortful control in the context of emotions, with a previous study showing an association between UF integrity and emotional control in children with traumatic brain injury (Johnson et al., 2011).

Results indicated a laterality effect, with only the right UF FA values being associated with emotion moderation scores. These findings are in line with previous research, which demonstrated lateralization of emotional processes. According to the valence hypothesis (Hellige, 2001), the processing of negative emotions preferentially engages the right side of the brain, whereas positive emotions are preferentially processed by the left side. This hypothesis is supported by functional MRI results showing participants are better at discriminating sad faces when visual stimuli are displayed in the left visual field (i.e., right hemisphere) and better at discriminating happy faces when displayed in the right visual field (i.e., left hemisphere; Adolphs et al., 2001; Rodway et al., 2003). The valence hypothesis is also supported by research showing that direct stimulation of the right amygdala induced unpleasant emotions, whereas stimulation of the left amygdala induced both pleasant and unpleasant emotions (Lanteaume et al., 2007). Further, differences between the two hemispheres in terms of volume and number of streamlines for UF subcomponents have been previously observed (Park et al., 2004; Hau et al., 2017), suggesting left-lateralized dorsolateral and right-lateralized ventromedial UF subcomponents.

Diffusion characteristics of any of the neonatal WM tracts studied here were not associated with social function and empathy factor scores. Social function was characterized by higher factor loadings for subscales evaluating social problems, and lower factor loadings for prosocial behavior, therefore higher social function scores reflected increased overall social difficulties. Better social function was associated with higher full-scale IQ, supporting previous literature highlighting the importance of cognitive development for successful social adaptation (Watson et al., 1999; Adolphs, 2001). These results could be important for translational research, as they suggest that an enhancement of children’s cognitive abilities could increase their understanding of social interactions (Soto-Icaza et al., 2015), leading to a more successful psychosocial adjustment. Furthermore, both social and emotional skills are thought to be intertwined with cognitive processes, with theoretical models postulating that emotions arise from evaluations of the goal relevance of a stimulus, and that other people are the most goal-relevant stimuli in one’s life (Olsson and Ochsner, 2008). Such framework could explain the inclusion of “SDQ emotional problems” in our social function factor.

Girls had higher empathy scores than boys, indicating a better ability to empathize with the experiences of others. Sex differences in empathy have been widely documented in previous research (Auyeung et al., 2009), and are thought to be reinforced by gender-based expectations of parents, teachers, and caregivers (Stern and Karraker, 1989).

Caveats of the present study include a lack of full-term control participants, which limits the generalizability of our findings only to those children born very preterm. In addition, the very preterm preschoolers who were followed-up in our study came from a relatively high socio-economic background (23.8% belonged to the least deprived IMD quintile) and had a higher mean full-scale IQ and lower “neonatal sickness index” scores compared with non-returners. Therefore, the current sample includes those children with a more favorable outcome and may not be representative of the overall ePrime sample and the wider very preterm population. Taking this into consideration, our results may be generalizable only to relatively high functioning very preterm children.

An added limitation of the current study is that dMRI was acquired using a b value of 750 s/mm^2^ with 32 non-collinear directions. Further, the diffusion metric FA is not suitable for modeling crossing fibers and is more susceptible to partial volume effects, limiting our ability to delineate the SLF in its entirety.

Another limitation is the omission of the cingulum in our analyses, as this tract has been associated with emotion processing and psychiatric symptomatology (Bubb et al., 2018). Cingulum tractography was not performed because TSA determines the tract skeleton by thinning the tract down to a medial surface, and therefore tube-like structures (such as the cingulum) are ill-suited for this methodology.

Results linking neonatal WM alterations with the tendency to overexpress negative emotions about oneself and the surrounding world in childhood, contributes to the growing body of research attempting to use an infant’s connectivity profile to predict its function in the future. This has been recently demonstrated by a study that used children’s connectivity fingerprints before they could read to predict their functional responses in a brain region hypothesized to be implicated in word recognition (i.e., the visual word form area) after they had learnt to read three years later (Saygin et al., 2016). The ability to predict typical and atypical patterns of emotional development would create opportunities for the early targeting of emerging emotional problems and their downstream consequences, including emotional disorders. This could be achieved through preventative therapies, such as emotion regulation training, that would help children to deal with potential stressors and enhance their mental health (World Health Organization, 2004; Brown et al., 2012).

## Synthesis

Reviewing Editor: Christie Fowler, University of California, Irvine Decisions are customarily a result of the Reviewing Editor and the peer reviewers coming together and discussing their recommendations until a consensus is reached. When revisions are invited, a fact-based synthesis statement explaining their decision and outlining what is needed to prepare a revision will be listed below. The following reviewer(s) agreed to reveal their identity: Aaron Mattfeld, Michael Yassa.

Both reviewers appreciated the changes incorporated in the revised version of the manuscript. In the consultation session, consensus was reached that the manuscript provides important findings that will be of interest for the field. However, a few minor points were raised, which will need to be addressed in a revised version of the manuscript.

1. In regards to the newly added methods at request of Reviewer #1. It would be good to clarify the protocol used for the TSA approach as it seems as though there is conflicting information. On line 174-175 the authors indicate that diffusion images were registered to a study-specific template for future analysis. Then on line 187-188 the authors indicate that diffusion images were then projected onto the skeletonized images containing the medial surface for each pathway of interest. It is unclear why the individual images need to be projected into skeleton space if the authors have already aligned them to a study-specific template.

2. In regards to point #4 and crossing fibers. The authors correctly indicate that they are limited in the number of directions as well as b-value at which the sequence was acquired. The authors also indicate previous difficulty in modeling crossing fibers (citing Pannek et al., 2012) in neonatal cohorts. While the sequence may be underpowered this does not necessarily limit the authors from selecting a reconstruction method that is designed to model crossing fibers. It is important to mention the limitation that FA, in general, is not suitable for modeling crossing fibers and is more susceptible to partial volume effects which may be responsible for the inability to model some pathways in their entirety.

3. Please make sure that Figure 3b depicting the factor loadings does not get cut-off in the final version of the document.

## Author Response

Synthesis of Reviews:

Computational Neuroscience Model Code Accessibility Comments for Author (Required):

N/A

Significance Statement Comments for Author (Required):

Appropriate

Comments on the Visual Abstract for Author (Required):

N/A

Synthesis Statement for Author (Required):

Both reviewers appreciated the changes incorporated in the revised version of the manuscript.

In the consultation session, consensus was reached that the manuscript provides important

findings that will be of interest for the field. However, a few minor points were raised, which

will need to be addressed in a revised version of the manuscript.

- We thank the editor for their positive comments, we appreciate the feedback and have

made the changes suggested as outlined below.

1. In regards to the newly added methods at request of Reviewer #1. It would be good to

clarify the protocol used for the TSA approach as it seems as though there is conflicting

information. On line 174-175 the authors indicate that diffusion images were registered to a

study-specific template for future analysis. Then on line 187-188 the authors indicate that

diffusion images were then projected onto the skeletonized images containing the medial

surface for each pathway of interest. It is unclear why the individual images need to be

projected into skeleton space if the authors have already aligned them to a study-specific

template.

- We have now clarified this in Methods 2.2.2, page 6. Projecting onto the skeleton after

registration serves a dimensionality reduction step for the statistical analysis as a way of

increasing sensitivity, though at the cost of specificity. This is described in detail in

section Statistical analysis on medial manifolds in the original TSA paper (Yushkevich et

al., 2008). Further, when the maximum FA value is projected onto the skeleton (similar to

TBSS), the projection step is used as an additional correction for residual misalignments

that may persist even after registration.

2. In regards to point #4 and crossing fibers. The authors correctly indicate that they are

limited in the number of directions as well as b-value at which the sequence was acquired.

The authors also indicate previous difficulty in modeling crossing fibers (citing Pannek et al.,

2012) in neonatal cohorts. While the sequence may be underpowered this does not

necessarily limit the authors from selecting a reconstruction method that is designed to model

crossing fibers. It is important to mention the limitation that FA, in general, is not suitable for

modeling crossing fibers and is more susceptible to partial volume effects which may be

responsible for the inability to model some pathways in their entirety.2

- Thank you. We agree it is important we make note of this limitation of FA and have

included this in our Discussion (please see page 16).

3. Please make sure that Figure 3b depicting the factor loadings does not get cut-off in the

final version of the document.

- Thank you for pointing this out, we will ensure this figure is not cut-off.

References

Yushkevich, P.A., Zhang, H., Simon, T.J., Gee, J.C., 2008. Structure-specific statistical mapping

of white matter tracts. Neuroimage 41, 448-461

## References

[B1] Adolphs R (2001) The neurobiology of social cognition. Curr Opin Neurobiol 11:231–239. 10.1016/s0959-4388(00)00202-6 11301245

[B2] Adolphs R, Jansari A, Tranel D (2001) Hemispheric perception of emotional valence from facial expressions. Neuropsychology 15:516–524. 11761041

[B3] Akaike H (1974) A new look at the statistical model identification. IEEE Trans Automat Contr 19:716–723. 10.1109/TAC.1974.1100705

[B4] Andersson JLR, Sotiropoulos SN (2016) An integrated approach to correction for off-resonance effects and subject movement in diffusion MR imaging. Neuroimage 125:1063–1078. 10.1016/j.neuroimage.2015.10.019 26481672 PMC4692656

[B5] Auyeung B, Wheelwright S, Allison C, Atkinson M, Samarawickrema N, Baron-Cohen S (2009) The children’s empathy quotient and systemizing quotient: sex differences in typical development and in autism spectrum conditions. J Autism Dev Disord 39:1509–1521. 10.1007/s10803-009-0772-x 19533317

[B6] Barnett ML, Tusor N, Ball G, Chew A, Falconer S, Aljabar P, Kimpton JA, Kennea N, Rutherford M, David Edwards A, Counsell SJ (2018) Exploring the multiple-hit hypothesis of preterm white matter damage using diffusion MRI. Neuroimage Clin 17:596–606. 10.1016/j.nicl.2017.11.017 29234596 PMC5716951

[B7] Belsky J, Friedman SL, Hsieh KH (2001) Testing a core emotion-regulation prediction: does early attentional persistence moderate the effect of infant negative emotionality on later development? Child Dev 72:123–133. 10.1111/1467-8624.00269 11280474

[B8] Brenner RG, Smyser CD, Lean RE, Kenley JK, Smyser TA, Cyr PEP, Shimony JS, Barch DM, Rogers CE (2021) Microstructure of the dorsal anterior cingulum bundle in very preterm neonates predicts the preterm behavioral phenotype at 5 years of age. Biol Psychiatry 89:433–442. 10.1016/j.biopsych.2020.06.015 32828528 PMC8064762

[B9] Brown CM, Copeland KA, Sucharew H, Kahn RS (2012) Social-emotional problems in preschool-aged children: opportunities for prevention and early intervention. Arch Pediatr Adolesc Med 166:926–932. 10.1001/archpediatrics.2012.793 22926145 PMC3578344

[B10] Bubb EJ, Metzler-Baddeley C, Aggleton JP (2018) The cingulum bundle: anatomy, function, and dysfunction. Neurosci Biobehav Rev 92:104–127. 10.1016/j.neubiorev.2018.05.008 29753752 PMC6090091

[B11] Calcagno V, de Mazancourt C (2010) Glmulti: an r package for easy automated model selection with (generalized) linear models. J Stat Softw 34:1–29.

[B12] Catani M, Dell’acqua F, Thiebaut de Schotten M (2013) A revised limbic system model for memory, emotion and behaviour. Neurosci Biobehav Rev 37:1724–1737. 10.1016/j.neubiorev.2013.07.001 23850593

[B13] Chen HF, Huang LL, Li HY, Qian Y, Yang D, Qing Z, Luo CM, Li MC, Zhang B, Xu Y (2020) Microstructural disruption of the right inferior fronto-occipital and inferior longitudinal fasciculus contributes to WMH-related cognitive impairment. CNS Neurosci Ther 26:576–588. 10.1111/cns.13283 31901155 PMC7163793

[B14] Constantino JN, Gruber CP (2012) Social responsiveness scale second edition (srs-2): manual. London: Western Psychological Services.

[B15] Costello AB, Osborne J (2005) Best practices in exploratory factor analysis: four recommendations for getting the most from your analysis. Practical Assess Res Eval 10:1–9.

[B16] Counsell SJ, Ball G, Edwards AD (2014) New imaging approaches to evaluate newborn brain injury and their role in predicting developmental disorders. Curr Opin Neurol 27:168–175. 10.1097/WCO.0000000000000073 24561870

[B17] Cullen KR, Klimes-Dougan B, Muetzel R, Mueller BA, Camchong J, Houri A, Kurma S, Lim KO (2010) Altered white matter microstructure in adolescents with major depression: a preliminary study. J Am Acad Child Adolesc Psychiatry 49:173–183.e171. 10.1097/00004583-201002000-00011 20215939 PMC2909686

[B18] Dalrymple KA, Gomez J, Duchaine B (2013) The dartmouth database of children’s faces: acquisition and validation of a new face stimulus set. PLoS One 8:e79131. 10.1371/journal.pone.0079131 24244434 PMC3828408

[B19] Digman JM (1990) Personality structure: emergence of the five-factor model. Annu Rev Psychol 41:417–440. 10.1146/annurev.ps.41.020190.002221

[B20] Dimitrova R, Pietsch M, Christiaens D, Ciarrusta J, Wolfers T, Batalle D, Hughes E, Hutter J, Cordero-Grande L, Price AN, Chew A, Falconer S, Vecchiato K, Steinweg JK, Carney O, Rutherford MA, Tournier JD, Counsell SJ, Marquand AF, Rueckert D, et al. (2020) Heterogeneity in brain microstructural development following preterm birth. Cereb Cortex 30:4800–4810. 10.1093/cercor/bhaa069 32306044 PMC7391275

[B21] Dodson CK, Travis KE, Ben-Shachar M, Feldman HM (2017) White matter microstructure of 6-year old children born preterm and full term. Neuroimage Clin 16:268–275. 10.1016/j.nicl.2017.08.005 28840098 PMC5558468

[B22] Duerden EG, Foong J, Chau V, Branson H, Poskitt KJ, Grunau RE, Synnes A, Zwicker JG, Miller SP (2015) Tract-based spatial statistics in preterm-born neonates predicts cognitive and motor outcomes at 18 months. AJNR Am J Neuroradiol 36:1565–1571. 10.3174/ajnr.A4312 25929880 PMC7964703

[B23] Eden AS, Schreiber J, Anwander A, Keuper K, Laeger I, Zwanzger P, Zwitserlood P, Kugel H, Dobel C (2015) Emotion regulation and trait anxiety are predicted by the microstructure of fibers between amygdala and prefrontal cortex. J Neurosci 35:6020–6027. 10.1523/JNEUROSCI.3659-14.2015 25878275 PMC6605169

[B24] Edwards AD, Redshaw ME, Kennea N, Rivero-Arias O, Gonzales-Cinca N, Nongena P, Ederies M, Falconer S, Chew A, Omar O, Hardy P, Harvey ME, Eddama O, Hayward N, Wurie J, Azzopardi D, Rutherford MA, Counsell S; ePrime Investigators (2018) Effect of mri on preterm infants and their families: a randomised trial with nested diagnostic and economic evaluation. Arch Dis Child Fetal Neonatal Ed 103:F15–F21. 10.1136/archdischild-2017-313102 28988160 PMC5750369

[B25] Eisenberg N, Cumberland A, Spinrad TL, Fabes RA, Shepard SA, Reiser M, Murphy BC, Losoya SH, Guthrie IK (2001) The relations of regulation and emotionality to children’s externalizing and internalizing problem behavior. Child Dev 72:1112–1134. 10.1111/1467-8624.00337 11480937

[B26] Fischi-Gómez E, Vasung L, Meskaldji DE, Lazeyras F, Borradori-Tolsa C, Hagmann P, Barisnikov K, Thiran JP, Hüppi PS (2015) Structural brain connectivity in school-age preterm infants provides evidence for impaired networks relevant for higher order cognitive skills and social cognition. Cereb Cortex 25:2793–2805. 10.1093/cercor/bhu073 24794920

[B27] Gao X, Maurer D (2009) Influence of intensity on children’s sensitivity to happy, sad, and fearful facial expressions. J Exp Child Psychol 102:503–521. 10.1016/j.jecp.2008.11.002 19124135

[B28] Goodman R (1997) The strengths and difficulties questionnaire: a research note. J Child Psychol Psychiatry 38:581–586. 10.1111/j.1469-7610.1997.tb01545.x 9255702

[B29] Hair JF (2009) Multivariate data analysis. Upper Saddle River: Prentice Hall.

[B30] Hanson JL, Knodt AR, Brigidi BD, Hariri AR (2015) Lower structural integrity of the uncinate fasciculus is associated with a history of child maltreatment and future psychological vulnerability to stress. Dev Psychopathol 27:1611–1619. 10.1017/S0954579415000978 26535947 PMC4698331

[B31] Hau J, Sarubbo S, Houde JC, Corsini F, Girard G, Deledalle C, Crivello F, Zago L, Mellet E, Jobard G, Joliot M, Mazoyer B, Tzourio-Mazoyer N, Descoteaux M, Petit L (2017) Revisiting the human uncinate fasciculus, its subcomponents and asymmetries with stem-based tractography and microdissection validation. Brain Struct Funct 222:1645–1662. 10.1007/s00429-016-1298-6 27581617

[B32] Hellige JB (2001) Hemispheric asymmetry: what’s right and what’s left. Cambridge: Harvard University Press.

[B33] Honey CJ, Sporns O, Cammoun L, Gigandet X, Thiran JP, Meuli R, Hagmann P (2009) Predicting human resting-state functional connectivity from structural connectivity. Proc Natl Acad Sci USA 106:2035–2040. 10.1073/pnas.0811168106 19188601 PMC2634800

[B34] Horning SM, Cornwell RE, Davis HP (2012) The recognition of facial expressions: an investigation of the influence of age and cognition. Neuropsychol Dev Cogn B Aging Neuropsychol Cogn 19:657–676. 10.1080/13825585.2011.645011 22372982

[B35] Jenkins LM, Barba A, Campbell M, Lamar M, Shankman SA, Leow AD, Ajilore O, Langenecker SA (2016) Shared white matter alterations across emotional disorders: a voxel-based meta-analysis of fractional anisotropy. Neuroimage Clin 12:1022–1034. 10.1016/j.nicl.2016.09.001 27995068 PMC5153602

[B36] Jenkinson M, Beckmann CF, Behrens TEJ, Woolrich MW, Smith SM (2012) Fsl. Neuroimage 62:782–790. 10.1016/j.neuroimage.2011.09.015 21979382

[B37] Johnson CP, Juranek J, Kramer LA, Prasad MR, Swank PR, Ewing-Cobbs L (2011) Predicting behavioral deficits in pediatric traumatic brain injury through uncinate fasciculus integrity. J Int Neuropsychol Soc 17:663–673. 10.1017/S1355617711000464 21492497 PMC3707392

[B38] Jones KM, Champion PR, Woodward LJ (2013) Social competence of preschool children born very preterm. Early Hum Dev 89:795–802. 10.1016/j.earlhumdev.2013.06.008 23870752 PMC4271316

[B39] Kessels RP, Montagne B, Hendriks AW, Perrett DI, de Haan EH (2014) Assessment of perception of morphed facial expressions using the emotion recognition task: normative data from healthy participants aged 8-75. J Neuropsychol 8:75–93. 10.1111/jnp.12009 23409767

[B40] Keunen K, Benders MJ, Leemans A, Fieret-Van Stam PC, Scholtens LH, Viergever MA, Kahn RS, Groenendaal F, de Vries LS, van den Heuvel MP (2017) White matter maturation in the neonatal brain is predictive of school age cognitive capacities in children born very preterm. Dev Med Child Neurol 59:939–946. 10.1111/dmcn.13487 28675542

[B41] Kleine I, Falconer S, Roth S, Counsell SJ, Redshaw M, Kennea N, Edwards AD, Nosarti C (2020) Early postnatal maternal trait anxiety is associated with the behavioural outcomes of children born preterm <33 weeks. J Psychiatr Res 131:160–168. 32977236 10.1016/j.jpsychires.2020.09.010 PMC7676467

[B42] Koshiyama D, Fukunaga M, Okada N, Morita K, Nemoto K, Yamashita F, Yamamori H, Yasuda Y, Matsumoto J, Fujimoto M, Kudo N, Azechi H, Watanabe Y, Kasai K, Hashimoto R (2020) Association between the superior longitudinal fasciculus and perceptual organization and working memory: a diffusion tensor imaging study. Neurosci Lett 738:135349. 10.1016/j.neulet.2020.135349 32889005

[B43] Lanteaume L, Khalfa S, Régis J, Marquis P, Chauvel P, Bartolomei F (2007) Emotion induction after direct intracerebral stimulations of human amygdala. Cereb Cortex 17:1307–1313. 10.1093/cercor/bhl041 16880223

[B44] Lautarescu A, Pecheva D, Nosarti C, Nihouarn J, Zhang H, Victor S, Craig M, Edwards AD, Counsell SJ (2020) Maternal prenatal stress is associated with altered uncinate fasciculus microstructure in premature neonates. Biol Psychiatry 87:559–569. 10.1016/j.biopsych.2019.08.010 31604519 PMC7016501

[B45] Montagna A, Nosarti C (2016) Socio-emotional development following very preterm birth: pathways to psychopathology. Front Psychol 7:80. 26903895 10.3389/fpsyg.2016.00080 PMC4751757

[B46] Mori S, Crain BJ, Chacko VP, van Zijl PC (1999) Three-dimensional tracking of axonal projections in the brain by magnetic resonance imaging. Ann Neurol 45:265–269. 10.1002/1531-8249(199902)45:2<265::AID-ANA21>3.0.CO;2-3 9989633

[B47] Mossad SI, Smith ML, Pang EW, Taylor MJ (2017) Neural correlates of “theory of mind” in very preterm born children. Hum Brain Mapp 38:5577–5589. 10.1002/hbm.23750 28766907 PMC6866839

[B48] Mullen KM, Vohr BR, Katz KH, Schneider KC, Lacadie C, Hampson M, Makuch RW, Reiss AL, Constable RT, Ment LR (2011) Preterm birth results in alterations in neural connectivity at age 16 years. Neuroimage 54:2563–2570. 10.1016/j.neuroimage.2010.11.019 21073965 PMC3020252

[B49] Nigg JT (2006) Temperament and developmental psychopathology. J Child Psychol Psychiatry 47:395–422. 10.1111/j.1469-7610.2006.01612.x 16492265

[B50] Noble KG, Korgaonkar MS, Grieve SM, Brickman AM (2013) Higher education is an age-independent predictor of white matter integrity and cognitive control in late adolescence. Dev Sci 16:653–664. 10.1111/desc.12077 24033571 PMC3775010

[B51] Olson IR, McCoy D, Klobusicky E, Ross LA (2013) Social cognition and the anterior temporal lobes: a review and theoretical framework. Soc Cogn Affect Neurosci 8:123–133. 10.1093/scan/nss119 23051902 PMC3575728

[B52] Olsson A, Ochsner KN (2008) The role of social cognition in emotion. Trends Cogn Sci 12:65–71. 10.1016/j.tics.2007.11.010 18178513

[B53] Park HJ, Westin CF, Kubicki M, Maier SE, Niznikiewicz M, Baer A, Frumin M, Kikinis R, Jolesz FA, McCarley RW, Shenton ME (2004) White matter hemisphere asymmetries in healthy subjects and in schizophrenia: a diffusion tensor MRI study. Neuroimage 23:213–223. 10.1016/j.neuroimage.2004.04.036 15325368 PMC2794419

[B54] Parkinson C, Wheatley T (2014) Relating anatomical and social connectivity: white matter microstructure predicts emotional empathy. Cereb Cortex 24:614–625. 10.1093/cercor/bhs347 23162046

[B55] Pecheva D, Yushkevich P, Batalle D, Hughes E, Aljabar P, Wurie J, Hajnal JV, Edwards AD, Alexander DC, Counsell SJ, Zhang H (2017) A tract-specific approach to assessing white matter in preterm infants. Neuroimage 157:675–694. 10.1016/j.neuroimage.2017.04.057 28457976 PMC5607355

[B56] Phan KL, Orlichenko A, Boyd E, Angstadt M, Coccaro EF, Liberzon I, Arfanakis K (2009) Preliminary evidence of white matter abnormality in the uncinate fasciculus in generalized social anxiety disorder. Biol Psychiatry 66:691–694. 10.1016/j.biopsych.2009.02.028 19362707 PMC2743779

[B57] Philippi CL, Mehta S, Grabowski T, Adolphs R, Rudrauf D (2009) Damage to association fiber tracts impairs recognition of the facial expression of emotion. J Neurosci 29:15089–15099. 10.1523/JNEUROSCI.0796-09.2009 19955360 PMC2819193

[B58] Putnam SP, Rothbart MK (2006) Development of short and very short forms of the children’s behavior questionnaire. J Pers Assess 87:102–112. 10.1207/s15327752jpa8701_09 16856791

[B59] Putnam SP, Gartstein MA, Rothbart MK (2006) Measurement of fine-grained aspects of toddler temperament: the early childhood behavior questionnaire. Infant Behav Dev 29:386–401. 10.1016/j.infbeh.2006.01.004 17138293 PMC4334385

[B60] Rieffe C, Ketelaar L, Wiefferink CH (2010) Assessing empathy in young children: construction and validation of an empathy questionnaire (emque). Pers Individ Dif 49:362–367. 10.1016/j.paid.2010.03.046

[B61] Riley JD, Franklin DL, Choi V, Kim RC, Binder DK, Cramer SC, Lin JJ (2010) Altered white matter integrity in temporal lobe epilepsy: association with cognitive and clinical profiles. Epilepsia 51:536–545. 10.1111/j.1528-1167.2009.02508.x 20132296 PMC2929974

[B62] Rodway P, Wright L, Hardie S (2003) The valence-specific laterality effect in free viewing conditions: the influence of sex, handedness, and response bias. Brain Cogn 53:452–463. 10.1016/s0278-2626(03)00217-3 14642295

[B63] Rogers CE, Anderson PJ, Thompson DK, Kidokoro H, Wallendorf M, Treyvaud K, Roberts G, Doyle LW, Neil JJ, Inder TE (2012) Regional cerebral development at term relates to school-age social-emotional development in very preterm children. J Am Acad Child Adolesc Psychiatry 51:181–191. 10.1016/j.jaac.2011.11.009 22265364 PMC3411187

[B64] Rogers CE, Sylvester CM, Mintz C, Kenley JK, Shimony JS, Barch DM, Smyser CD (2017) Neonatal amygdala functional connectivity at rest in healthy and preterm infants and early internalizing symptoms. J Am Acad Child Adolesc Psychiatry 56:157–166. 10.1016/j.jaac.2016.11.005 28117062 PMC5302247

[B65] Rothbart MK, Bates JE (1998) Temperament. In: Handbook of child psychology: social, emotional, and personality development, Ed 5, pp 105–176. Hoboken: Wiley, Inc.

[B66] Rothbart MK, Ellis LK, Rueda MR, Posner MI (2003) Developing mechanisms of temperamental effortful control. J Pers 71:1113–1143. 10.1111/1467-6494.7106009 14633060

[B67] Salvan P, Froudist Walsh S, Allin MP, Walshe M, Murray RM, Bhattacharyya S, McGuire PK, Williams SC, Nosarti C (2014) Road work on memory lane–functional and structural alterations to the learning and memory circuit in adults born very preterm. Neuroimage 102:152–161. 10.1016/j.neuroimage.2013.12.031 24368264

[B68] Saygin ZM, Osher DE, Norton ES, Youssoufian DA, Beach SD, Feather J, Gaab N, Gabrieli JD, Kanwisher N (2016) Connectivity precedes function in the development of the visual word form area. Nat Neurosci 19:1250–1255. 10.1038/nn.4354 27500407 PMC5003691

[B69] Smith SM (2002) Fast robust automated brain extraction. Hum Brain Mapp 17:143–155. 10.1002/hbm.10062 12391568 PMC6871816

[B70] Smith SM, Jenkinson M, Johansen-Berg H, Rueckert D, Nichols TE, Mackay CE, Watkins KE, Ciccarelli O, Cader MZ, Matthews PM, Behrens TE (2006) Tract-based spatial statistics: voxelwise analysis of multi-subject diffusion data. Neuroimage 31:1487–1505. 10.1016/j.neuroimage.2006.02.024 16624579

[B71] Soto-Icaza P, Aboitiz F, Billeke P (2015) Development of social skills in children: neural and behavioral evidence for the elaboration of cognitive models. Front Neurosci 9:333. 10.3389/fnins.2015.00333 26483621 PMC4586412

[B72] Spittle AJ, Treyvaud K, Doyle LW, Roberts G, Lee KJ, Inder TE, Cheong JLY, Hunt RW, Newnham CA, Anderson PJ (2009) Early emergence of behavior and social-emotional problems in very preterm infants. J Am Acad Child Adolesc Psychiatry 48:909–918. 10.1097/CHI.0b013e3181af8235 19633579

[B73] Stern M, Karraker KH (1989) Sex stereotyping of infants: a review of gender labeling studies. Sex Roles 20:501–522. 10.1007/BF00288198

[B74] Sylvester CM, Smyser CD, Smyser T, Kenley J, Ackerman JJ Jr, Shimony JS, Petersen SE, Rogers CE (2018) Cortical functional connectivity evident after birth and behavioral inhibition at age 2. Am J Psychiatry 175:180–187. 10.1176/appi.ajp.2017.17010018 28774192 PMC5794627

[B75] Thomson KC, Richardson CG, Gadermann AM, Emerson SD, Shoveller J, Guhn M (2019) Association of childhood social-emotional functioning profiles at school entry with early-onset mental health conditions. JAMA Netw Open 2:e186694. 10.1001/jamanetworkopen.2018.6694 30646194 PMC6324314

[B76] Travis KE, Adams JN, Ben-Shachar M, Feldman HM (2015) Decreased and increased anisotropy along major cerebral white matter tracts in preterm children and adolescents. PLoS One 10:e0142860. 10.1371/journal.pone.0142860 26560745 PMC4641645

[B77] Unger A, Alm KH, Collins JA, O’Leary JM, Olson IR (2016) Variation in white matter connectivity predicts the ability to remember faces and discriminate their emotions. J Int Neuropsychol Soc 22:180–190. 10.1017/S1355617715001009 26888615 PMC5494205

[B78] Urbain C, Sato J, Hammill C, Duerden EG, Taylor MJ (2019) Converging function, structure, and behavioural features of emotion regulation in very preterm children. Hum Brain Mapp 40:3385–3397. 10.1002/hbm.24604 31056820 PMC6865470

[B79] Vollmer B, Lundequist A, Mårtensson G, Nagy Z, Lagercrantz H, Smedler AC, Forssberg H (2017) Correlation between white matter microstructure and executive functions suggests early developmental influence on long fibre tracts in preterm born adolescents. PLoS One 12:e0178893. 10.1371/journal.pone.0178893 28594884 PMC5464584

[B80] Von Der Heide RJ, Skipper LM, Klobusicky E, Olson IR (2013) Dissecting the uncinate fasciculus: disorders, controversies and a hypothesis. Brain 136:1692–1707. 10.1093/brain/awt094 23649697 PMC3673595

[B81] Wakana S, Caprihan A, Panzenboeck MM, Fallon JH, Perry M, Gollub RL, Hua K, Zhang J, Jiang H, Dubey P, Blitz A, van Zijl P, Mori S (2007) Reproducibility of quantitative tractography methods applied to cerebral white matter. NeuroImage 36:630–644. 10.1016/j.neuroimage.2007.02.049 17481925 PMC2350213

[B82] Wang W, Qian S, Liu K, Li B, Li M, Xin K, Sun G (2016) Reduced white matter integrity and its correlation with clinical symptom in first-episode, treatment-naive generalized anxiety disorder. Behav Brain Res 314:159–164. 10.1016/j.bbr.2016.08.017 27515289

[B83] Watson AC, Nixon CL, Wilson A, Capage L (1999) Social interaction skills and theory of mind in young children. Dev Psychol 35:386–391. 10.1037//0012-1649.35.2.386 10082009

[B84] Wechsler D (2012) Wechsler preschool and primary scale of intelligence, Ed 4. San Antonio: The Psychological Corporation.

[B85] Witt A, Theurel A, Tolsa CB, Lejeune F, Fernandes L, de Jonge L. v H, Monnier M, Graz MB, Barisnikov K, Gentaz E, Hüppi PS (2014) Emotional and effortful control abilities in 42-month-old very preterm and full-term children. Early Hum Dev 90:565–569. 10.1016/j.earlhumdev.2014.07.008 25105752

[B86] Woodward LJ, Lu Z, Morris AR, Healey DM (2017) Preschool self regulation predicts later mental health and educational achievement in very preterm and typically developing children. Clin Neuropsychol 31:404–422. 10.1080/13854046.2016.1251614 27801620

[B87] World Health Organization (2004) Prevention of mental disorders: effective interventions and policy options: summary report. Geneva: World Health Organization.

[B88] Young JM, Vandewouw MM, Morgan BR, Smith ML, Sled JG, Taylor MJ (2018) Altered white matter development in children born very preterm. Brain Struct Funct 223:2129–2141. 10.1007/s00429-018-1614-4 29380120

[B89] Yushkevich PA, Zhang HG (2013) Deformable modeling using a 3d boundary representation with quadratic constraints on the branching structure of the blum skeleton. Inf Process Med Imaging 23:280–291. 10.1007/978-3-642-38868-2_24 24683976 PMC3974205

[B90] Yushkevich PA, Zhang H, Simon TJ, Gee JC (2008) Structure-specific statistical mapping of white matter tracts. Neuroimage 41:448–461. 10.1016/j.neuroimage.2008.01.013 18407524 PMC2519052

[B91] Zhang H, Yushkevich PA, Alexander DC, Gee JC (2006) Deformable registration of diffusion tensor MR images with explicit orientation optimization. Med Image Anal 10:764–785. 10.1016/j.media.2006.06.004 16899392

[B92] Zheng KZ, Wang HN, Liu J, Xi YB, Li L, Zhang X, Li JM, Yin H, Tan QR, Lu HB, Li BJ (2018) Incapacity to control emotion in major depression may arise from disrupted white matter integrity and ofc-amygdala inhibition. CNS Neurosci Ther 24:1053–1062. 10.1111/cns.12800 29368421 PMC6489955

